# Mental health professionals’ experiences with shared decision-making for patients with psychotic disorders: a qualitative study

**DOI:** 10.1186/s12913-020-05949-1

**Published:** 2020-11-27

**Authors:** Espen W. Haugom, Bjørn Stensrud, Gro Beston, Torleif Ruud, Anne S. Landheim

**Affiliations:** 1 Norwegian National Advisory Unit on Concurrent Substance Abuse and Mental Health Disorders, Innlandet Hospital Trust, P. B 104, 2381 Brumunddal, Norway; 2 Norwegian Centre for Addiction Research (SERAF), Institute of Clinical Medicine, University of Oslo, Blindern, Oslo, Norway; 3 Innlandet Hospital Trust, Division of Mental Health, P.B 104, 2381 Brumunddal, Norway; 4 Mental Health Services, Akershus University Hospital, Lørenskog, Norway; 5 Institute of Clinical Medicine, University of Oslo, Blindern, Oslo, Norway; 6 Department of Public Health, Inland Norway University of Applied Sciences, Elverum, Norway

**Keywords:** Shared decision-making, Mental health care, Mental health professionals, Psychotic disorders

## Abstract

**Background:**

Shared decision-making (SDM) is a process whereby clinicians and patients work together to select treatments based on both the patient’s preferences and clinical evidence. Although patients with psychotic disorders want to participate more in decisions regarding their care, they have limited opportunities to do so because of various barriers. Knowing about health professionals’ experiences with SDM is important toward achieving successful implementation. The study aim was to describe and explore health professionals’ SDM experiences with patients with psychotic disorders.

**Methods:**

Three focus group interviews were conducted, with a total of 18 health professionals who work at one of three Norwegian community mental health centres where patients with psychotic disorders are treated. We applied a descriptive and exploratory approach using qualitative content analysis.

**Results:**

Health professionals primarily understand the SDM concept to mean giving patients information and presenting them with a choice between different antipsychotic medications. Among the barriers to SDM, they emphasized that patients with psychosis have a limited understanding of their health situation and that time is needed to build trust and alliances. Health professionals mainly understand patients with psychotic disorders as a group with limited abilities to make their own decisions. They also described the concept of SDM with little consideration of presenting different treatment options. Psychological or social interventions were often presented as complementary to antipsychotic medications, rather than as an alternative to them.

**Conclusion:**

Health professionals’ understanding of SDM is inconsistent with the definition commonly used in the literature. They consider patients with psychotic disorders to have limited abilities to participate in decisions regarding their own treatment. These findings suggest that health professionals need more theoretical and practical training in SDM.

## Background

Shared decision-making (SDM) is often defined as “a process in which clinicians and patients work together to select tests, treatments, management or support packages, based on clinical evidence and the patient’s informed preferences” [1]. SDM involves presenting information about the patient’s health issue and treatment options, acknowledging their values and preferences, discussing pros and cons, and considering the patients’ abilities and self-efficacy. Further, when the health professional presents their recommendation, the SDM practice is to check and clarify the patient’s understanding, make or explicitly defer a decision, and arrange follow-up [2]. On a continuum from clinician-led to patient-led decision-making, SDM practice is considered an intermediate model of collaboration [3, 4].

User involvement and voluntariness have been two of the most important political guidelines for mental health work over recent decades [5, 6]. SDM is closely connected to user involvement, as it aims to strengthen the patient’s decisional position [7]. In recent decades, SDM has garnered growing interest, and it continues to be promoted in an effort to improve the quality of services [1]. However, implementation in routine mental health care has been limited [3] and even less accessible to those with severe mental illness [4], despite their wanting to participate more in decisions regarding their care [8–10].

A cross-sectional survey of 846 outpatients found that having schizophrenia is a risk factor for being involved with lower levels of SDM practice [11]. A review found that SDM in mental health care occurs less often than patients desire, and that health professionals may approach patients with schizophrenia with a directive style or even the use of pressure [12]. The same review showed that health care providers see patients’ reduced decisional capacity as a major barrier to implementing SDM in mental health care [12].

A structured questionnaire of 352 psychiatrists explored their use of SDM in schizophrenia treatment, revealing that SDM was seen as useful for patients who exhibited insight, were well-informed, or explicitly demanded participation [13]. However, SDM was not judged to be an appropriate approach in cases of potentially reduced decisional capacity, and the psychiatrists expressed doubts about whether they could accept these patients as competent partners in medical decisions about issues such as hospitalization or antipsychotic medication [13]. Other qualitative studies of consultant psychiatrists’ experiences with prescribing antipsychotic medications also support the notion that patients’ decisional capacity can be a barrier to SDM [14, 15].

A 2017 amendment to the Norwegian mental health act states that patients may refuse treatment if they are considered competent to consent [16]. Being competent means they understand the information relevant to their specific health situation, recognize the factors in their unique situation, can see the consequences of various treatment options for them personally, can reason about relevant information in weighing various treatment options, and have the ability to communicate their choice [17]. The amendment indicates that patients who regain their decisional capacity may, at any time, terminate a treatment which has been initiated. However, this does not apply if there is an immediate and serious danger to the patient’s life or to the life or health of others [16].

Involving service users is a decisive component of contemporary health care [6, 18] and a broad national movement to implement SDM is underway in Norway [19]. The national health portal recently published a decision aid for patients with a psychotic disorder [20]. The hospital trusts are required to ensure that mental health care patients are, as far as it is reasonable, able to choose between different treatment options, including mental health treatment without medication [21].

Further research on health professionals’ experiences with practicing SDM with patients with psychotic disorders is needed, so that we may better understand how to implement this practice. The study aim was to describe and explore health professionals’ understanding of, and experiences with, SDM, and the barriers to using SDM with patients with psychotic disorders.

## Methods

### Design

This qualitative focus group study of health professionals used a descriptive and exploratory design [22], with an inductive approach [23] following Graneheim and Lundman’s qualitative content analysis [24]. The analysis focused on subject and context, emphasizing differences between and similarities within codes and categories [23]. The study’s epistemological point of view was within a phenomenological and hermeneutic tradition.

### Implementation support and training

This study is an independent part of a larger project investigating the implementation of four different evidence-based practices (EBP) for psychosis treatment: antipsychotic medication, family interventions, somatic health care, and illness management and recovery [25].

For the larger project, all participating community mental health centres (CMHC) and hospital departments selected two EBP to implement. They were randomized to receive implementation support for one EBP and no support for the other. The degree to which each of the EBP were implemented was assessed four times over 18 months using a five-point fidelity scale (1 = poor fidelity, 5 = high fidelity). Each CMHC was offered in-person implementation support biweekly for 6 months and then monthly for an additional 12 months. The aim of the support was to help health professionals identify and overcome barriers, and to engage them in implementing the assigned EBP [25].

One of the 15 subscales in the fidelity measurement tool for antipsychotic medication is the use of SDM [26]. Thus, the study’s implementation support included SDM practice and 14 other components. Eight sites were randomized to receive implementation support for the antipsychotic medication EBP. These sites were offered a one-day workshop with Norwegian experts on antipsychotic medication management. An average of nine leaders and clinicians from each site participated. SDM was one of the workshop topics. The mean fidelity score on the SDM subscale for the eight sites was 1.63 (standard deviation 0.92) after 18 months. Five sites scored 1, one site scored 2, and two sites scored 3.

### Recruitment and setting

Health professionals were recruited from CMHCs which were offered implementation support for the antipsychotic medication EBP. To achieve a sample with representative variation in experiences with SDM, we identified and contacted four CMHCs with a range in the degree of their fidelity ratings for SDM implementation. We provided brief information about the study, followed by an invitation to present the study. Two CMHCs accepted at that stage, after which a fifth CMHC was invited, and agreed, to participate. A total of 18 health professionals from the three CMHCs participated. Recruitment took place from November 2018 to May 2019. Participants from the first CMHC were recruited from an inpatient unit which also offered outpatient care. From the second CMHC, health professionals from one outpatient unit participated. Participants from the third CMHC were recruited from four units, two outpatient and two inpatient.

The study was carried out within three different Norwegian health trusts: i.e., two in the east and one in the north. These health trusts’ services for patients with psychotic disorders include acute psychiatric units, units for the treatment of psychotic disorders, and different outpatient care options. The participating hospital trusts’ catchment areas include cities, smaller towns, and rural regions. The municipalities in these areas also provide mental health services. The participating CMHCs’ SDM fidelity scores were 1, 3, and 3 after 18 months, respectively, indicating a range from no implementation to a moderate degree of implementation.

### Participants

The head of each CMHC decided which health professionals to invite to participate in the study, based on the criterion that they have experience using decision-making practice with patients with psychotic disorders. Eleven women and seven men participated. Nine of the 18 participants worked exclusively in outpatient treatment, six worked in both inpatient and outpatient treatment, and three worked exclusively in inpatient treatment. The three participants who worked exclusively with inpatient treatment were psychiatrists. Seven of these 18 health professionals had participated in the workshop previously: one from focus group 1, four from focus group 2, and two from focus group 3. Table 1 shows an overview of the participants.

**Table 1:** Participant characteristics

	Focus group 1	Focus group 2	Focus group 3	Total	Outpatient treatment	Inpatient treatment	Inpatient and outpatient
Psychiatrist	1	6	1	8	3	3	2
Psychologist	2			2	2		
Medical doctor	1			1	1		
Mental health nurse	2			2	2		
Nurse		1	2	3	1		2
Social educator			2	2			2
Total	6	7	5	18	9	3	6

In Norwegian mental health care, psychiatrists and medical doctors are responsible for prescribing medications, while nurses and social educators are responsible for administering them. Nurses, social educators, psychiatrists, and psychologists each have individual treatment responsibilities in outpatient care. However, according to the Mental Health Care Act, a psychiatrist or clinical psychologist is responsible for legally based decisions [16].

### Data collection

We conducted three focus group interviews. This method was considered appropriate to the study goals, as interactions within focus groups can highlight participants’ experiences and provide a framework of understanding [27]. The interviews were conducted using a thematic guide designed by the research group, which includes the third author who had prior user experience. The thematic guide developed for this study is provided as Additional File 1. Each interview was begun with an open-ended question asking participants to share their SDM experiences with patients with psychotic disorders. The interviewer then asked questions about their understanding of the SDM concept, whether and how they practice SDM, and their understanding of inhibitors/promoters of SDM. The questions were all open-ended and aimed to allow the participants to freely share their experiences. The interviews were conducted during May and June 2019. We conducted the 60–90-min interviews in a meeting room at the participating CMHC. The first and third authors co-led each interview: e.g., the latter paid special attention to the dialogue between the participants and took notes. Both asked for more details when necessary to clarify the participant’s statements.

### Data analysis

After each focus group interview, the first and third authors summarized their impressions and reflections. Interviews were audio recorded and transcribed verbatim by the first author. Information about each participant’s profession and whether they worked in inpatient or outpatient treatment was included in the transcript, as health professionals from different contexts participated. The materials were analysed as a whole, and the unit of analysis was the transcribed text from the three interviews [24]. The first author read the transcribed interviews several times to gain a familiarity with the material. Meaning units were identified and labelled by code (i.e., words, sentences, or paragraphs relating to each other through their content and context) [24]. Condensing involved writing a shorter text while preserving the core meaning. The first, second, third, and last authors discussed and compared the codes for differences and similarities before they were sorted into categories describing the content on a manifest level [23]. At this time, the analysis was discussed among a group of Ph.D. students during a qualitative methods course meeting led by a teacher at the university with comprehensive experience with qualitative methods. The preliminary results were also presented to colleagues. The feedback from these two groups was included in further analysis and compared to our preliminary results. This led to a broader understanding of the material. Two overarching themes unifying the threads running through the categories were identified through interpretation of the latent content. Themes and categories were compared with the interview data to ensure that they covered the use of SDM as stated by the participants [24]. Finally, the categories were presented using representative quotes from the participants to anchor the content. In selecting the quotes, we emphasized solid anchorage in the text and differentiating between categories [23, 24].

The first author had a background as a social educator with several years working in acute psychiatry. The second author was a psychiatric nurse with extensive experience treating patients with schizophrenia and other psychoses. The author with prior user experience contributed a different perspective to the analysis process, challenging the first author’s position and resulting in a reflexive process that provided a more meaningful understanding of the participants’ experiences [28]. The fourth author is professor emeritus, a clinical psychiatrist and a mental health services researcher with experiences from doing many studies, including as principal investigator for the multicenter study which includes the current study. The last author is sociologist and has been working with health services research in mental health care for many years. The categorization of findings was supported using NVivo 11 pro.

### Ethical considerations

The participants were informed both verbally and in writing about the study before they signed their voluntary, informed consent. Each interview was anonymized by removing any identifying information, to ensure participant confidentiality. Data were stored on a secure research server approved by the Data Protection Officer at the Innlandet Hospital Trust. The ethical principles of the World Medical Association’s 2013 Declaration of Helsinki were followed. The study was approved by the Regional Committee for Medical and Health Research Ethics (REC South-East, reg. no 2015/2169). The study was also approved by the Data Protection Officer at each participating health trust.

## Results

The analysis resulted in two main themes. The first theme, *the SDM concept*, consisted of two categories related to health professionals’ experiences with understanding and practicing SDM. The second theme, *barriers to SDM*, consisted of two categories related to health professionals’ experiences with barriers to SDM.

### Theme: the SDM concept

#### Providing information

Several participants stated that SDM involves providing patients with information. They also said that they mainly provide information about the patient’s diagnosis and state of health. Some reported only giving verbal information, while others said they also provide a written brochure. Some participants reported using a fill-in form or a board on which they draw shapes, while others use different websites to show videos. One participant expressed that it is important to make the information easily understandable and non-threatening, using language that the patient understands. Others said psycho-education is useful for informing patients about their disorder:

I think psycho-education is important in relation to a psychosis diagnosis. I find that people who have an established diagnosis really know very little about their disorder. And that it is rather mysterious in a way. (Nurse, outpatient care)

Some participants noted that they provide information about different treatment options to allow patients to make choices in line with their values. The same participants expressed that it is most important that patients receive the information they need to make choices and that their voice is heard:

The essence of it, as I see it, is that the patient’s voice should be heard. The person should be able to express his opinion. It should be given weight in the conversation. (Psychiatrist 1 [P1], in- and outpatient care)

Several participants reported using SDM in their practice. They said that patients in both out- and inpatient facilities are given the opportunity to make joint decisions. One participant stated that SDM implies both providing information as well as one can and receiving the best information possible. Another said that SDM is always used.

#### Medication treatment decisions

Many participants said that SDM is a question of whether a patient should have antipsychotic medication. One psychiatrist expressed this as:

What I’ve said before, when I’ve been asked that question, is that the question is often whether the patient should have medicine or not. And the next thing is that if they have used medications before, then they may have an idea about what type of medicine they want or don’t want. Apart from that, they haven’t the least idea about what kind of medicine they want. If they haven’t taken medicine before, then they have no idea whether to take this medicine or that medicine. It’s only [a matter of whether they should have] medicine or not. (P1, in- and outpatient care)

Some participants stated that they provide general information about different types of antipsychotic medication and alternative forms of administration. They said that patients are asked about their experiences with antipsychotic medication. Some psychiatrists said that their choice of medication is based on written information and their own clinical experience. Other psychiatrists reported that patients are willing to try a medicine if they present it to them. Several participants expressed their belief that health professionals know what is best for the patient:

You should never have to get so ill that you actually have to go to hospital again, but then you have to have a good dialogue with us. So that I can make the choices for you that are the best in terms of dosages and quantities and types of medication. (P2, outpatient care)

Several participants said that it is important to them to monitor any side effects and that they are willing to collaborate with the patient to choose an alternative medication if side effects are experienced. Some noted that it is particularly important to monitor weight gain closely, and that they had focused too little on this side effect in the past. Some participants mentioned that it is important to inform patients of the consequences of discontinuing a medicine.

Some participants stated that they refer patients to a treatment alternative if they do not want to take antipsychotic medication. Several participants said that other treatment options are offered in addition to medication treatment (e.g., family therapy, cognitive therapy, job support). Some participants said they wish their workplace offered more than medical treatment and counselling. Others reported finding it challenging to present patients with alternatives because they feel there are no clear distinctions between different methods and treatment approaches, such as when deciding whether to base treatment on a psychodynamic, cognitive, or behavioural approach. These participants stated that instead of presenting such alternatives, they focus on the patients’ treatment goals and what the patient wants to work on.

### Theme: barriers to SDM

#### Trust and therapeutic alliance take time

The participants said that they could spend months or even years learning what is important to some patients because it takes a long time for their patients to feel confident enough to share their wishes with a therapist:

It’s quite true what’s been said here now, the time factor is very important, you spend time building an alliance and trust. And the quality of SDM, or the quality of the choices made, will increase when you trust the person who’s suggesting something. So that’s why I support this idea that the time factor is absolutely crucial. (P2, outpatient care)

Several participants conveyed that patience is needed to help patients to open up and talk about symptoms that they had been too distrustful to mention previously. According to several participants, as the patient’s suspicions decrease and their confidence in the relationship increases, a firmer basis for co-operation on treatment options develops.

Several participants shared that a therapeutic alliance needs to be built to access information about what the patient wants, and to help them:

That’s what I think is most important. To build an alliance between you and your patients. (P3, in- and outpatient care)

Some participants said that a good therapeutic alliance is important for building mutual trust between the patient and professional. Therapist continuity was mentioned by some as important for building a good alliance with the patient. Some participants said that dialogue is a useful tool for discovering what is most important to the patient.

Although most participants agreed that time and therapeutic alliance are needed to identify what is important to the patient, a few participants took a different view. One participant spoke about using the alliance to communicate the importance of a treatment:

It’s an important part of treatment, or one of the most important parts of treatment, is to build a therapeutic alliance and at least try to increase the patient’s insight into the disease and then let him know that medication can be a really important part of treatment. (P4, inpatient care)

Another participant stated:

We need to get an alliance that results in the patient agreeing to a little more than they might have expected, so that I can feel reassured and so that they can be confident that we are doing things pretty much in the right way. (P2, outpatient care)

This person talked about using the alliance to persuade the patient to accept more of the treatment the therapists wanted to offer.

#### Limited understanding of one’s own situation

Almost all the participating psychiatrists and several other participants reported experiencing challenges with using SDM with patients with psychotic disorders whom they felt had a limited understanding of the consequences of the disorder for their lives. Some of the participants said early in the interview that they aimed to come to an agreement with the patient. Later in the interview, the same participants reported that, in many situations, they had to make the decision for the patient because of the patient’s limited understanding. Some participants justified this based on the patient’s ambivalence; in situations in which it was difficult to get a clear answer from the patient, the professionals must make the choice. Others said that the sickest patients often refuse treatment. This was illustrated by a dialogue among the psychiatrists P3 (in- and outpatient care), P5 (inpatient care), and P6 (outpatient care):

P3: “The sickest people say no to whatever we offer them.”

P5: “Yes, they do.”

P3: “In psychiatry, the ones who need the most treatment generally say no. That’s my experience.”

P6: “They say no because they’re paranoid and suspicious.”

P5: “They’re in very bad shape.”

Several participants found it particularly challenging if their patient was psychotic and said there are situations in which they, as therapists, must make the decisions. One participant also stated that SDM is not necessarily possible beginning at the patient’s first contact with the therapist:

You’re psychotic, coming on strong with some paranoia. So you’re not capable of making decisions here and now, but later on we can talk about SDM. (P5, inpatient care)

The participants described situations with violent or suicidal patients as especially challenging. A couple of participants described these as demanding situations in which they must think carefully about whether to ignore the patient’s wants. Others said it is difficult to arrive at a common solution if the therapist disagrees with the patient. Some felt that SDM is not suitable for all patients.

I don’t think SDM fits all types of patients and diagnoses. You have to be a bit realistic. (P3, in- and outpatient care)

This participant later continued:

It’s good that those who can decide are allowed to decide, but not everybody. The ones who are very sick, they can’t decide. (P3, in- and outpatient care)

Another participant emphasized that the therapist cannot let patient involvement go so far that the therapist just gives in:

But by law, our patients have the right to user participation. Then I’m thinking about what you said, and I said something about it just now, if we were to go whole hog, and now I’m talking about the ones who are here voluntarily, then we’d be giving way too much if we didn’t use our own expertise and kind of try to influence [the decision] in one direction or another. If it’s too much, it’s informal coercion, if it’s too little, we’re just giving way. You have to find the right balance. (Nurse, outpatient care)

Although most participants stated that they had to make treatment decisions for patients, especially those who are sickest, some had different views:

Choice means choice. Isn’t that the way life is for many people? Suddenly you’re affected and then you make a choice, and maybe in fact it was a stupid choice. (Social educator, in- and outpatient care)

Some participants said that patients with psychotic disorders must be fully respected. Health professionals must be willing to let these patients make a choice and live with the consequences of that choice, even if they disagree.

## Discussion

This study shows that when treating patients with psychosis, health professionals limit their SDM approach to giving their patients information about their diagnosis and health situation, and that SDM is predominantly a matter of choosing between antipsychotic medication types. Psychological and social treatment options are usually offered as a supplement, not an alternative, to medication treatment. The study also shows that health professionals feel it is more difficult to practice SDM with patients who have a limited understanding of their own situation and when they need considerable time to build trust and a therapeutic alliance.

### The understanding and practice of SDM

These mental health professionals emphasized that they provide patients with information about their condition, a core area of SDM [12]. They try to make this information easily comprehensible and non-threatening, such as with psycho-education, which can facilitate SDM [12]. The professionals are thus practising basic elements of SDM. However, as we can see in Table 2 the study also shows that it is unclear how far this is followed up with other elements of SDM, e.g., such as presenting different treatment options, considering the patient’s values and preferences, or discussing the advantages and disadvantages of alternatives [2].

**Table 2:** How the findings reflect essential elements of SDM as described by Makoul et al. [2]

Essential elements of SDM	Findings in this study
Define/explain problem	The participants inform the patients about the diagnosis and state of health.
Present options	The participants inform the patients to some degree about different antipsychotic medications. If psychological and social treatment options are presented, it is usually as a supplement, not as an alternative, to medication.
Discuss pro/cons	Usually discussed after prescribing an antipsychotic medication and not as part of a SDM process before making the treatment choice.
Patient values/preferences	Some participants mention the importance of providing care in line with patients’ values, but our results do not support that this is participants’ everyday practice.
Discuss patient ability/self-efficacy	The findings do not give a clear answer as to what degree the participants discuss patient ability/self-efficacy.
Doctor knowledge/recommendation	Some participants present an antipsychotic medication to the patients. However, our findings do not support that it is a recommendation based on a SDM process where different options and their pros/cons have been discussed in light of the patients’ values.
Check/clarify understanding	The findings are unclear regarding to what degree the health professionals’ check the patients’ understanding.
Make or explicitly defer decision	The findings suggest that health professionals often make the decision due to the patients’ limited understanding of their own situation. This is a practice more based on a clinical-led model than on SDM.
Arrange follow-up	The participants describe using time to identify what is important for the patients suggesting that they follow-up decisions over time. However, it is unclear how and to what extent this is implemented as part of an overall SDM process.

The study shows that, to a limited extent, health professionals report collaborating with patients on treatment choices. Simultaneously, several participants believe that they are using SDM. This shows that the health professionals quoted herein have a different, and limited, understanding of the concept of SDM. This is consistent with previous research showing that health professionals believe they are using SDM, while observer-based outcomes indicate that it has not yet been implemented in clinical practice [29].

That the health professional participants in this study describe SDM as being largely about medication treatment choices suggests that a medical understanding of treatment predominates. This is surprising, since several of the participants are psychologists and other professionals with backgrounds in health and social care. The participants explained that psychological, social, and other treatment interventions may be offered—as a supplement, not an alternative—to medication treatment. That these participating CMHC staff were intended to implement an EBP on antipsychotic medication may have directed their focus towards medication treatment. However, in recent years both the Norwegian Ministry of Health and service user organizations have strongly focused on non-pharmaceutical treatment alternatives [21]. Thus, the participants should have emphasized alternatives more than was done herein.

That half of those in our sample were psychiatrists and medical doctors may help explain the limited extent to which they offer alternative interventions. Another interview study found that the prevailing attitude among psychiatrists in community services is that antipsychotic medication is an important aspect of treating patients with psychotic disorders [14]. Psychiatrists often have a leading role on treatment teams, which may also have given them a more dominant role in the focus group discussions, potentially leading to pressure for a consensus on medication approaches. The other half of the sample was divided among different professions, with only two psychologists participating. This may have made it challenging for others to express different experiences, possibly explaining why they did not describe any clear alternative to the medication approach.

The participating health professionals were offered training and implementation guidance. However, that they report practicing only limited aspects of SDM might also be explained by the fact that 11 of the 18 participants did not attend the training course and therefore lacked the requisite knowledge. Another explanation may be that a one-day training course in which SDM is only one of several topics is insufficient. The implementation guidance was provided over 18 months and SDM was one of 15 implementation areas. This may have made it challenging to focus adequately on SDM and contributed to their limited practice of this approach.

It is important to interpret the participants’ understanding of medical treatment within the context of current guidelines, which state that treatment with antipsychotic medication is recommended and that psychological interventions are most effective when combined with medication [30]. This helps explain why the medical treatment approach apparently predominates. Some participants stated that they would like to offer alternatives to medical treatment and counselling, which is consistent with the emphasis by service user organizations and health authorities on the importance of patient choice between different treatment options, including medication-free services [21].

The study findings show that patients with psychotic disorders have limited involvement in SDM where medication-free services are an alternative. Some health professionals do inform their patients about different types of antipsychotic medication and explore the patient’s previous experiences. Other patients may only be presented with one type of antipsychotic medication and are scarcely involved in collaboration. At the same time, several health professionals expressed a willingness to collaborate on choosing another medication if the patient has experienced side effects. This is important because the decision to risk metabolic syndrome, tardive dyskinesia, or reduced libido in exchange for symptom relief is not only a medical, but a personal matter [31]. However, a practice consistent with the intentions of SDM implies that risks and disadvantages (e.g., side effects) are discussed before a choice is made [2].

### Barriers to practising SDM

Limited time is one of the most commonly reported barriers to SDM implementation [32]. The professionals in our study also described time as a barrier. They say it can take months to years to build a therapeutic alliance that enables SDM. They consider this duration necessary to reduce suspicion and learn what is important to the patient. No previous findings have supported the notion that SDM increases consultation time [33]. Some studies show that SDM can be implemented in mental health without increasing the time involved. For example, Deegan [34] showed that despite time constraints, technology and peer support can facilitate SDM. Despite this, decisions in mental health care can be more complex and take longer than in other fields of medicine [12]. Decisions are thus not necessarily made in one consultation, but over several consultations. This underlines the importance of being patient and taking time to build an alliance based on trust, which can lead to a positive dialogue that elicits the patient’s values and preferences.

The therapeutic alliance highlights the need for good co-operation when using SDM [35]. This can be challenging when patients have limited decision-making capacity, as described by the participants herein. In such cases, co-operation is often abandoned in favour of a more asymmetric relationship [14]. This study shows that although the therapeutic alliance is important, it can also be used to make the patient agree with the therapist. For example, the participants described how they use their alliance to make the patient accept more of the treatment recommended by the health professional.

Participants described patients’ limited understandings of their own situations as a barrier to SDM, which concurs with previous research [12]. For this reason, health professionals believe they must make the treatment decision for the patient. This may be seen as a practice based more on a clinician-led model than on SDM, a view consistent with previous research showing that SDM is infrequently used in mental health care [3].

Health professionals sometimes justify making unilateral decision by vaguely stating that the patients are very sick. Alternatively, they state more specifically that the patients are psychotic, violent, or suicidal. In some of these situations, SDM will not necessarily take place during the initial contact with the therapist. However, it is interesting that the assessment of patients’ limited understanding of their situation persists and is described by the participants as being a major barrier to SDM. This is contradicted by research findings showing that the majority of people with schizophrenia are considered to have adequate decision-making capacity [36]. This is particularly interesting because several of the health professionals we interviewed work in an outpatient setting with patients who mainly live in the community. In comparison with inpatients, one would expect these outpatients to have better functioning and be more likely to be judged capable of making decisions. SDM should therefore be used more with this group of patients.

Nevertheless, participants state that they must make decisions for their patients in many contexts. One explanation for this may be the generalization that emerged in the interviews based on patients whose functioning is lowest because it was difficult to focus the interviews on outpatients with higher levels of functioning. The interviews often contain descriptions of experiences with patients in crisis and in which hospitalization must be considered. Some participants worked only in inpatient care (*n* = 3), while some worked in both in- and outpatient care (*n* = 6); thus, they would naturally have experience with sicker patients. Another explanation is that health professionals’ views on the treatment of patients with psychotic disorders are based on a more clinician-led model. It is important to note here that amendments to the Mental Health Act in 2017 gave patients greater self-determination [16]. If patients have decision-making capacity, they have a right to make choices with which clinicians disagree, and a right to make poor choices. The findings of this study suggest a potential for greater reflection on the significance of the law amendments, in terms of values and practicalities.

Although several health professionals in this study stated that they do not give patients any responsibility in the decision-making process, some also expressed that one should be more willing to let patients decide, even if the decisions can be considered poor choices. The latter is in line with health policy guidelines on increased service user participation and autonomy [5]. However, it is also important to realize that some patients with acute psychosis will not have decision-making capacity. Decision-making capacity is contextual, it can vary over time and must be understood in relation to each specific decision to be made [37]. This study suggests a potential for increasing the focus on the meaning of decision-making capacity in the implementation of SDM.

### Strengths and limitations

A strength of this study is that the interactions among the focus group participants provided detailed descriptions of their experiences [27, 38]. The involvement of a researcher with prior user experience in most phases of the study is a strength that may have enhanced the quality of the study [39]. Four researchers were involved in the analysis, which was presented both to colleagues and within a qualitative methods course. This is a strength because it ensured reflexive processes in which we sought out positions that challenged our preunderstandings.

There are also several potential study limitations. First, half the participants were psychiatrists or medical doctors. However, two of the groups only included one psychiatrist. In these two groups, the psychiatrists spoke slightly more than the other participants did. Most of the participants promoted their views, both the psychiatrists and other professions. However, we cannot guaranty that the psychiatrists – with a leading role in the team - did not influence group consensus and thus silenced dissenting voices [27]. This may be a limitation of our recruitment strategy. The moderators tried to shift the attention to participants that spoke less by seeking eye contact and if necessary asking direct questions. Second, including participants from the same clinical team may have influenced the dynamics in the focus group as norms in the teams may have silenced participants with dissenting experiences. Further, synergism in the group may have allowed the participants to strengthen their experiences of how difficult it is to practice SDM for patients with a psychotic disorder and contributed to more critical comments [27]. Third, the study was conducted in a limited geographical area and thus may represent only local practices. Fourth, we only included health professionals’ perspectives, a few of whom contributed less than others. Fifth, we included those working in both inpatient and outpatient units, although we tried to minimize this issue by contextualizing the participants’ experiences (i.e., identified each quote with the participant’s profession and workplace). Sixth, we do not know how many of our participants’ patients were in involuntary versus voluntary treatment, which may have influenced the interview conversations. Seventh, we only interviewed participants working at CMHCs where SDM had not, or only moderately, been implemented, possibly explaining why they spoke more about difficulties with SDM and less about success factors.

Finally, further interviews may have provided additional perspectives. However, in applying a qualitative design, we did not intend to generalize the participants’ experiences. The intention was to elucidate the phenomenon through good descriptions from the participants. The third interview confirmed previous themes, without providing new perspectives. That other studies [12] have found some of the same results may strengthen the validity of our findings beyond the participants in this study. We suggest that the results may be relevant to those working in mental health services that treat patients with psychotic disorders.

## Conclusions

This study shows that health professionals’ inclusion of patients with psychotic disorders in decisions regarding their treatment is limited. Health professionals describe an understanding of SDM that is relatively limited to providing information about the patient’s health situation and medication treatment choices. SDM is primarily not practiced to the degree of collaboration between health professionals and patients that its definition intends. This suggests that the current SDM practice is not in line with guidelines by health authorities.

This study shows that more comprehensive SDM training is needed, with focuses on values, attitudes, how to carry out SDM in clinical practice, and, potentially, communication skills. The findings suggest that implementation support should be aimed more precisely at SDM, to increase the chances of health professionals adopting the approach.

Additional research addressing health professionals’ use of SDM with patients with psychotic disorders will be needed. Such research should focus on the factors that lead to health professionals to make greater use of SDM. Additional research may include investigating both health professionals’ and society’s understandings of their roles and responsibilities in empowering this patient group. Future research might also more closely examine how decision-making capacity is understood and assessed in carrying out SDM for patients with psychotic disorders.

## Supplementary Information

**Additional file 1:.** Thematic guide.

## Data Availability

The qualitative data material or parts of the data may be considered available from the first author Espen W. Haugom upon reasonable request. Contact details: Espen.Woldsengen.Haugom@Sykehuset-Innlandet.no

## Abbreviations

CMHC: Community mental health centres
EBP: Evidence-based practices
SDM: Shared decision-making

## References

[1] Coulter A, Collins A (2011). Making shared decision-making a reality: no decision about me, without me.

[2] Makoul G, Clayman ML (2006). An integrative model of shared decision making in medical encounters. Patient Educ Couns.

[3] Slade M (2017). Implementing shared decision making in routine mental health care. World Psychiatry.

[4] Hamann J, Heres S (2014). Adapting shared decision making for individuals with severe mental illness. Psychiatr Serv.

[5] Helse- og omsorgsdepartementet [Norwegian Ministry of Health and Care Services] (2015). Nasjonal helse- og sykehusplan [National health and hospital plan] (2016–2019). Meld St 11 (2015–2016).

[6] Omeni E, Barnes M, MacDonald D, Crawford M, Rose D (2014). Service user involvement: impact and participation: a survey of service user and staff perspectives. BMC Health Serv Res.

[7] Charles C, Gafni A, Whelan T (1997). Shared decision-making in the medical encounter: what does it mean? (or it takes at least two to tango). Soc Sci Med.

[8] Adams JR, Drake RE, Wolford GL (2007). Shared decision-making preferences of people with severe mental illness. Psychiatr Serv.

[9] Dahlqvist Jonsson P, Schon UK, Rosenberg D, Sandlund M, Svedberg P (2015). Service users’ experiences of participation in decision making in mental health services. J Psychiatr Ment Health Nurs.

[10] Stensrud B, Hoyer G, Granerud A, Landheim AS (2015). “Life on hold”: a qualitative study of patient experiences with outpatient commitment in two Norwegian counties. Issues Ment Health Nurs.

[11] De las Cuevas C, Penate W (2014). To what extent psychiatric patients feel involved in decision making about their mental health care? Relationships with socio-demographic, clinical, and psychological variables. Acta Neuropsychiatr.

[12] Beitinger R, Kissling W, Hamann J (2014). Trends and perspectives of shared decision-making in schizophrenia and related disorders. Curr Opin Psychiatry.

[13] Hamann J, Mendel R, Cohen R, Heres S, Ziegler M, Buhner M (2009). Psychiatrists’ use of shared decision making in the treatment of schizophrenia: patient characteristics and decision topics. Psychiatr Serv.

[14] Seale C, Chaplin R, Lelliott P, Quirk A (2006). Sharing decisions in consultations involving anti-psychotic medication: a qualitative study of psychiatrists’ experiences. Soc Sci Med.

[15] Shepherd A, Shorthouse O, Gask L (2014). Consultant psychiatrists’ experiences of and attitudes towards shared decision making in antipsychotic prescribing, a qualitative study. BMC Psychiatry.

[16] Psykisk helsevernloven (1999). The Norwegian Mental Health Act.

[17] Grisso T, Appelbaum P (1998). Assessing competence to consent to treatment. A guide for physicians and other health professionals.

[18] Pasient-og brukerrettighetsloven (1999). The Norwegian Patient Rights Act.

[19] Kasper J, Lager AR, Rumpsfeld M, Kienlin S, Smestad KH, Brathen T (2017). Status report from Norway: implementation of patient involvement in Norwegian health care. Z Evid Fortbild Qual Gesundhwes.

[20] The National Health Portal [Internet]. (2019). Available from: https://minhelse.helsenorge.no/samvalg/verktoy/psykose/mitt-valg. Accessed 18 September 2019.

[21] Helse- og omsorgsdepartementet [Norwegian Ministry of Health and Care Services] (2015). Oppdragsdokument for 2015 [Assignment letter for 2015 to the Regional Health Authorities].

[22] Brink PJ, Wood MJ (1997). Advanced design in nursing research.

[23] Graneheim UH, Lindgren BM, Lundman B (2017). Methodological challenges in qualitative content analysis: a discussion paper. Nurse Educ Today.

[24] Graneheim UH, Lundman B (2004). Qualitative content analysis in nursing research: concepts, procedures and measures to achieve trustworthiness. Nurse Educ Today.

[25] ClinicalTrials.gov [Internet]. Bethesda (MD): National Library of Medicine (US). Identifier: NCT03271242 Implementation of National Guidelines for Treatment of Psychoses; 2017. Available from: https://clinicaltrials.gov/ct2/show/NCT03271242. Accessed 5 Feb 2020.

[26] Ruud T, Drivenes K, Drake RE, Haaland VO, Landers M, Stensrud B, et al. The antipsychotic medication management Fidelity scale: psychometric properties. Admin Pol Ment Health. 2020. 10.1007/s10488-020-01018-1. 10.1007/s10488-020-01018-1 PMC7547997 32030595

[27] Kitzinger J (1995). Qualitative research. Introducing focus groups. BMJ..

[28] Veseth M, Binder PE, Borg M, Davidson L (2017). Collaborating to stay open and aware: service user involvement in mental health research as an aid in reflexivity. Nord Psychol.

[29] Legare F, Thompson-Leduc P (2014). Twelve myths about shared decision making. Patient Educ Couns.

[30] National Institute for Health and Care Excellence (2014). Psychosis and schizophrenia in adults: prevention and management [Internet].

[31] Drake RE, Deegan PE (2009). Shared decision making is an ethical imperative. Psychiatr Serv.

[32] Legare F, Ratte S, Gravel K, Graham ID (2008). Barriers and facilitators to implementing shared decision-making in clinical practice: update of a systematic review of health professionals’ perceptions. Patient Educ Couns.

[33] Duncan E, Best C, Hagen S. Shared decision making interventions for people with mental health conditions. Cochrane Database Syst Rev. 2010:CD007297. 10.1002/14651858.CD007297.pub2 PMC7209977 20091628

[34] Deegan PE (2010). A web application to support recovery and shared decision making in psychiatric medication clinics. Psychiatr Rehabil J.

[35] Truglio-Londrigan M, Slyer JT, Singleton JK, Worral P (2012). A qualitative systematic review of internal and external influences on shared decision-making in all health care settings. JBI Libr Syst Rev.

[36] Jeste DV, Depp CA, Palmer BW (2006). Magnitude of impairment in decisional capacity in people with schizophrenia compared to normal subjects: an overview. Schizophr Bull.

[37] Ruissen AM, Widdershoven GA, Meynen G, Abma TA, van Balkom AJ (2012). A systematic review of the literature about competence and poor insight. Acta Psychiatr Scand.

[38] Cote-Arsenault D, Morrison-Beedy D (1999). Practical advice for planning and conducting focus groups. Nurs Res.

[39] Mjosund NH, Eriksson M, Espnes GA, Haaland-Overby M, Jensen SL, Norheim I (2017). Service user involvement enhanced the research quality in a study using interpretative phenomenological analysis - the power of multiple perspectives. J Adv Nurs.

