# Supplementary material for: Mental health professionals’ experiences with shared decision-making for patients with psychotic disorders: a qualitative study
Source: BMC Health Serv Res. 2020 Nov 27;20:1093. doi: 10.1186/s12913-020-05949-1 (PMC7694931; doi:10.1186/s12913-020-05949-1)
Supplement: Supplementary file 1 — Additional file 1:. Thematic guide. [file 12913_2020_5949_MOESM1_ESM.pdf]

Shared decision-making for patients with a psychotic disorder: Thematic guide for focus groups with health professionals

Main theme
Shared decision-making experiences with patients with psychotic disorders.

Sub-theme 1
Understanding of the shared decision-making concept.

Sub-theme 2
Whether and how they practice shared decision-making.

Sub-theme 3
Advantages versus disadvantages of practicing shared decision-making.

Sub-theme 4
Inhibitors and promoters with shared decision-making.
